# Next‐Generation Surgery: Integrating Artificial Intelligence, Genetic Technologies, Bioengineering and Rehabilitation Into Modern Practices

**DOI:** 10.1002/EXP.20240417

**Published:** 2026-05-28

**Authors:** Dengxiong Li, Jie Wang, Zhipeng Wang, Ruicheng Wu, Zhouting Tuo, Fanglin Shao, Wenjing Ge, Ziyu Shu, Yubo Yang, Dilinaer Wusiman, Qingxin Yu, Luxia Ye, Alisha Pati‐Alam, Facai Zhang, Koo Han Yoo, Susan Heavey, William C. Cho, Dechao Feng

**Affiliations:** ^1^ Department of Urology The First Affiliated Hospital of Zhejiang Chinese Medical University (Zhejiang Provincial Hospital of Chinese Medicine) Hangzhou Zhejiang Province China; ^2^ Department of Urology Institute of Urology West China Hospital Sichuan University Chengdu China; ^3^ Department of Urology Sichuan Provincial People's Hospital University of Electronic Science and Technology of China Chengdu China; ^4^ Department of Urology Tianjin Institute of Urology The Second Hospital of Tianjin Medical University Tianjin China; ^5^ Department of Rehabilitation The Affiliated Hospital of Southwest Medical University Luzhou P.R. China; ^6^ Department of Clinical Neurosciences University of Cambridge Cambridge Biomedical Campus Cambridge UK; ^7^ Department of Earth Science and Engineering Imperial College London London UK; ^8^ Joint International Research Laboratory of Green Buildings and Built Environments (Ministry of Education) Chongqing University Chongqing China; ^9^ Department of Urology Three Gorges Hospital Chongqing University Wanzhou Chongqing China; ^10^ Department of Comparative Pathobiology College of Veterinary Medicine Indiana USA; ^11^ Purdue Institute for Cancer Research Purdue University West Lafayette Indiana USA; ^12^ Department of Pathology Zhejiang University School of Medicine Research Unit of Intelligence Classification of Tumor Pathology and Precision Therapy Chinese Academy of Medical Sciences Hangzhou Zhejiang China; ^13^ Department of Pathology Peking Union Medical College Hospital Chinese Academy of Medical Sciences and Peking Union Medical College Beijing China; ^14^ Department of Public Research Platform Taizhou Hospital of Zhejiang Province Affiliated to Wenzhou Medical University Linhai China; ^15^ Division of Surgery & Interventional Science University College London London UK; ^16^ Urology & Nephrology Center Department of Urology, Clinical Medicine Research Institute Zhejiang Provincial People's Hospital(Affiliated People's Hospital), Hangzhou Medical College Hangzhou Zhejiang China; ^17^ Department of Urology Kyung Hee University Seoul South Korea; ^18^ Department of Clinical Oncology Queen Elizabeth Hospital Hong Kong China

**Keywords:** artificial intelligence, bioengineering, clinical practices, genetic technologies, next‐generation surgery, rehabilitation

## Abstract

Surgery has undergone a transformation in recent decades, from open operations to minimally invasive surgery. Significant advancements in technologies like robotic and minimally invasive techniques have led to better outcomes. However, simply enhancing anatomical knowledge and surgical skills has left the field of surgery stuck in a rut. Challenges like cost, training, patient safety, ethics, and global disparities persist. In the knowledge‐explosion era, surgical methods have been advanced by the integration of multidisciplinary cutting‐edge technologies like artificial intelligence (AI), genetic technologies, bioengineering, and elaborative rehabilitation scheme. Surgeons can more effectively and efficiently handle complicated clinical difficulties, optimize surgical operations, and provide individualized therapies by utilizing the combined power of these technologies, paving the way for reshaping contemporary surgical practices. Here, we overview how these key technological advancements have brought about the advent of “next‐generation surgery.”

## Graphical abstract

This graphic abstract depicts the convergence of artificial intelligence, genetic technologies, bioengineering, and rehabilitation as the core pillars of next‐generation surgery. These domains collectively enhance preoperative planning, intraoperative precision, tissue reconstruction, and postoperative recovery, driving a paradigm shift toward personalized, intelligent, and patient‐centered surgical care.

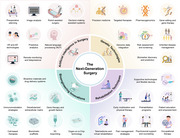

## 1 Introduction

Surgery is on the verge of a technological revolution, with cutting‐edge techniques changing established practices [1]. From antiquity through the Middle Ages, surgery remained primarily open procedures performed with rudimentary tools following high mortality rates. The 19th century breakthroughs in anesthesia and antisepsis established modern surgical foundations, while 20th‐century advances in medical imaging and life support enabled complex procedures like organ transplantation. Contemporary surgery integrates minimally invasive techniques, robotic systems, artificial intelligence (AI) navigation, and precision medicine, driving the field toward intelligent, personalized, and remote‐controlled approaches. There are concepts and techniques proposed to improve surgical skills and treatment outcomes [2]. These concepts and techniques include minimally invasive surgery, robotic‐assisted surgery, augmented reality (AR) and virtual reality (VR) technologies, enhanced recovery after surgery (ERAS) protocols, telemedicine and remote surgery, tissue engineering and regenerative medicine and simulation‐based training, which also play significant roles in improving and advancing surgical procedures [3, 4]. In addition, surgeons increasingly specialize in specific areas, such as neurosurgery, cardiothoracic surgery, orthopedic surgery, urology surgery and so on [5]. This specialization leads to deeper expertise and more targeted treatments. Interdisciplinary collaboration is also more common. Surgeons frequently collaborate with other medical professionals, including radiology, oncology, and anesthesia, to give complete care to patients [6, 7]. Multidisciplinary teams contribute to more effective treatment strategies and outcomes. Moreover, access to surgical care varies worldwide, with some areas lacking suitable facilities, equipment, and qualified workers [8]. Efforts are underway to increase global access to critical surgical treatments, particularly in low‐ and middle‐income nations.

However, surgery is a rapidly evolving field that faces challenges. Advanced surgical procedures and technologies can be costly, which presents issues for healthcare organizations, particularly in resource‐constrained areas [8]. Many patients continue to face major financial and accessibility barriers to surgical care. In addition, surgeons need to undergo extensive training and continuous education to maintain proficiency and stay up to date with innovations [9]. It is imperative to guarantee that surgeons have access to adequate training opportunities and resources, particularly in developing fields [10]. Access to timely and high‐quality surgical care is still unevenly distributed across and within nations, with rural and marginalized populations frequently encountering more obstacles in this regard. Reducing these disparities calls for coordinated efforts at the infrastructure, funding, and policy levels. In terms of patient safety, surgical operations are inherently risky due to the possibility of bleeding, problems from anesthesia, and infection, even with modern technological developments [11]. It is a never‐ending challenge to ensure patient safety through strict protocols, standardized practices, and quality improvement activities. On the other hand, careful thought is needed to preserve patient autonomy, beneficence, and justice when it comes to ethical conundrums in surgery, such as end‐of‐life decisions, organ transplantation, and experimental treatments [8, 12].

Overall, even though surgery has significantly improved patient outcomes and quality of life, successive efforts are required to address these issues and guarantee that patients have fair access to surgical care. Breakthroughs in surgical technology have transformed operations from focusing solely on preoperative preparation and intraoperative procedures into a systematic continuum spanning from outpatient care to complete postoperative recovery. This evolution demands surgeons' comprehensive understanding of next‐generation surgical approaches to advance the field. Accordingly, this article examines potentially transformative technologies and discoveries, including AI, genetic technology, bioengineering, and rehabilitation, that may significantly reshape future surgical practice.

## 2 Artificial Intelligence in Surgery

AI proves to be a potent ally in the field of surgery, utilizing autonomous systems, predictive analytics, and image recognition to enhance human talents [13, 14]. From preoperative planning to intraoperative decision‐making and postoperative care, AI algorithms use big data to improve surgical results, reduce risks, and increase patient safety [15, 16].

### 2.1 Preoperative Planning

AI algorithms help surgeons with preoperative planning by analyzing patient data such as test results, electronic health records, and medical imaging. This aids in figuring out possible issues, improving surgical techniques, and comprehending the anatomy of the patient. For instance, in the emergency department, significant time is often spent on preoperative tests, electronic health records, and medical imaging [17]. Thus, critical information may be overlooked due to the urgency of patient cases. A hybrid neural network‐based AI model could predict whether trauma patients require specialized surgical intervention within 12 h of hospital arrival, aiding clinicians in timely decision‐making [18]. Regarding routine surgery, AI can short the time of flap design in breast reconstruction by analyzing deep inferior epigastric perforator artery in CT data [19]. With the assistance of AI, surgical procedures can become safer and more efficient [20]. Therefore, AI can assist doctors in determining surgical necessity and optimizing operative schedules. This facilitates enhanced preoperative preparation for patients while expanding clinical decision‐making options. Particularly in emergency settings, such AI‐supported triage helps prevent the oversight of surgical patients.

### 2.2 Image Analysis

Medical images such as magnetic resonance imaging, computed tomography scans, and X‐rays can be accurately analyzed by AI algorithms with astonishing precision [21, 22]. In order to help surgeons make educated judgments during procedures, AI can help evaluate these images to identify anomalies, tumor margins, and crucial structures. For instance, AI analyzes preoperative CT scans of lung cancer patients to determine the presence of lymph node metastasis. This assists in identifying key areas and the quantities of lymph nodes to be resected during surgery [23]. Mei et al. [24] developed an AI model using CT urography images to predict perirenal fat adhesion in renal cell carcinoma, improving surgical accuracy and efficacy. Moreover, AI‐driven preoperative MRI analysis can evaluate the clinical characteristics of rectal cancer before operation, including extramural vascular invasion, lymph node metastasis, and T‐stage classification [25]. Additionally, AI‐integrated imaging can assess the tumor microenvironment, thereby predicting malignancy potential and treatment response [26]. These findings suggest that AI may leverage imaging data to provide critical clinical information for surgical planning and perioperative preparation, thereby establishing a robust foundation for comprehensive patient care throughout the treatment cycle.

### 2.3 Robot‐Assisted Surgery

AI‐driven surgical robots, like the da Vinci Surgical System, provide doctors more accuracy and dexterity while performing minimally invasive treatments. These robots improve surgical outcomes and shorten patient recovery times by filtering hand tremors, scaling down movements, and providing 3D visualization [27]. In the endoscopy field, endoscopic retrieval of esophageal squamous cell carcinoma assisted by AI demonstrates higher sensitivity and specificity compared with the control group, without adverse event observed [28]. Another multicenter RCT also demonstrated that AI‐assisted endoscopy significantly improved the detection rate of early esophageal squamous cell carcinoma, reduced missed diagnoses, and maintained acceptable safety profiles [29]. Although AI assistance can significantly improve surgical precision and reduce procedural difficulty, current AI‐assisted surgical robots remain incapable of replacing human surgeons to operate independently. This limitation exists because each patient's anatomical structure and pathological alterations are unique, frequently requiring intraoperative judgment to determine surgical margins and preserve normal physiological structures. An erroneous decision could lead to surgical failure and life‐threatening consequences. Furthermore, liability issues regarding unexpected accidents caused by autonomous AI‐assisted surgical robots remain unresolved. Therefore, current AI systems are not yet capable of independent surgical operation.

### 2.4 Decision‐Making Assistant Systems

DuCRring procedures, surgeons can receive real‐time guidance from AI‐based decision support systems. These systems provide recommendations on the best surgical techniques, instrument selection, and possible dangers based on the analysis of data from multiple sources, such as imaging devices, surgical instruments, and patient vitals [30]. In an 80‐year‐old woman diagnosed with pancreatic cancer, the surgeon utilized SYNAPSE VINCENT, a 3D image analysis‐based deep learning algorithm, for intraoperative navigation during partial pancreatic tumor resection [31]. The patient presented with lesions close to the pancreatic head, posing surgical challenges. This technology aids surgical personnel by providing anatomical guidance, displaying the thickness, volume, and precise location of the resecting pancreas, and facilitating real‐time information sharing within the surgical team. Ultimately, guided by this system, the surgical team successfully excised the tumor, preserving pancreatic tissue, with the frozen section confirming negative surgical margins. This demonstrates the significant role of integrating AI algorithms and imaging intraoperatively. Similarly, Chen et al. [32] developed an AI laparoscopic exploration system that enabled real‐time intraoperative detection of abdominal metastases during gastric cancer procedures. The system demonstrated comparable performance to novice surgeons in identifying abdominal metastases, with superior capability in detecting subtle and occult lesions. These demonstrate AI's potential to offer evidence‐based guidance for individualized surgical decision‐making.

### 2.5 VR and AR Technologies

Surgeons may practice surgical methods, model difficult procedures, and view patient anatomy in three dimensions with the help of AI‐powered VR and AR technologies. These virtual experiences reduce errors during actual surgeries, improve spatial awareness, and augment surgical training. In a renal cancer clinical study, combining the da Vinci surgical robot with 3D virtual models based on convolutional neural networks for robot‐assisted partial nephrectomy aids surgeons in avoiding blood vessels, excising tumors, and preserving renal units [33]. This illustrates the potential practical applications of VR and AR in surgery and patient education.

### 2.6 Predictive Analytics

AI algorithms are able to anticipate surgical results, postoperative complications, and patient recovery trajectories by analyzing large volumes of patient data [34, 35]. Surgeons can thus better tailor treatment regimens, reduce risks, and streamline patient care processes. For example, AI can automatically score pathological tissue stains from prostate cancer patients to predict survival prognosis [36]. Consequently, physicians can develop personalized postoperative treatment and follow‐up plans based on patient prognosis, particularly drug responses, thereby enhancing therapeutic outcomes and optimizing comprehensive patient management.

### 2.7 Natural Language Processing (NLP)

NLP approaches help extract and analyze information from unstructured clinical notes, research papers, and medical literature. Surgeons can use NLP‐powered tools to retrieve relevant clinical knowledge, stay current with research findings, and make evidence‐based decisions. Noord et al. reported that TLDR, an AI model, can read and summarize the content of medical papers in a sentence [37]. This function provides busy surgeons with summaries of the latest developments in the field and offers solution to intraoperative challenges through AI literature retrieval.

### 2.8 Remote Monitoring and Telepresence

With AI‐enabled telepresence systems, surgeons can remotely observe procedures, guide less experienced colleagues, and cooperate with diverse teams across geographic borders. This improves access to specialized care and encourages knowledge sharing among surgeons worldwide. Doctors and patients can guide patient diagnosis and treatment through remote conversations [38]. Remote surgery is also being gradually conducted and has been successful [39]. Therefore, AI‐assisted summarization of patient conditions by doctors and remote surgical guidance among doctors is highly anticipated direction. However, we need to notice that a surgical robot is the basis of remote surgery. While the costs of surgical robot procurement and utilization will ultimately be borne by patients, this does not necessarily increase expenses for all patients in underdeveloped or developing regions. Remote surgery should be reserved for patients whose local surgical teams lack capability or experience, rather than for routine surgeries. For these selected surgeries, remote surgery offers two key advantages: (1) eliminating the need for patients to travel abroad or domestically for specialized surgeries; and (2) reducing associated travel, accommodation, and follow‐up expenses. Although the direct medical costs may increase, the overall treatment expenditure could potentially decrease when considering the entire care cycle—a hypothesis requiring further clinical validation. Regardless, AI‐enhanced remote surgery provides underdeveloped regions with access to advanced medical care, representing a valuable area for exploration and development.

While AI has enormous potential for improving surgical practice, it is critical to address issues such as data privacy, regulatory compliance, and ethical considerations to ensure its safe and responsible integration into clinical processes. As technology advances, AI has the potential to improve surgery by improving patient outcomes, boosting surgical innovation, and pushing the frontiers of what is physically achievable.

## 3 Genetic Technologies Revolutionizing Surgical Interventions

The emergence of genetic technologies heralds a new era of precision medicine, in which surgical procedures are tailored to each patient's particular genetic composition. Genomic sequencing, gene editing, and pharmacogenomics enable surgeons to interpret hereditary predispositions, tailor treatment regimens and reduce adverse effects, ushering in a new era of personalized surgical care [40]. Genetic technologies are revolutionizing surgical operations in a variety of ways, most notably by delivering personalized insights into patients' genetic makeup and illness risk [41].

### 3.1 Precision Medicine

Genetic testing enables surgeons to customize treatment schedules based on a patient's genetic profile [42]. For instance, mutations in the CDKN2A significantly increase the risk of familial melanoma. Consequently, screening for the CDKN2A mutation allows surgeons to determine a patient's susceptibility to familial melanoma and recommend more frequent skin examinations and early surgical interventions [43, 44]. Surgeons can predict susceptibility to specific diseases, assess drug responses, and personalize treatment strategies to maximize efficacy while minimizing side effects by analyzing an individual's genetic variations.

### 3.2 Risk Assessment and Screening

Genetic screening detects people who are at high risk of developing hereditary disorders like breast cancer, bladder cancer, and cardiovascular disease [45, 46]. Surgeons can use these data to implement preventive strategies, recommend screening procedures, and perform prophylactic surgeries as needed to reduce disease load and enhance patient outcomes. For example, mutations in the BRCA1 and BRCA2 substantially increase the risk of breast and ovarian cancer in patients [47, 48]. Individuals with BRCA1/2 mutations may choose prophylactic mastectomy, prophylactic oophorectomy, or more intensive monitoring strategies [49]. Furthermore, aortic dissection and other related aortic diseases often have a genetic basis, including mutations in the FBN1 associated with Marfan Syndrome [50, 51]. This proactive approach can manage and minimize the need for emergency surgery, optimizing treatment pathways and patient outcomes. This strategy enables early‐stage disease control, decreasing both physical and psychological harm to patients and reducing associated economic and societal burdens.

### 3.3 Pharmacogenomics

Pharmacogenomic testing investigates genetic variations that affect drug metabolism and reaction [52]. For example, mutations in the VKORC1 and CYP2C9 significantly influence warfarin metabolism. This genetic testing enables surgeons to select the appropriate anticoagulant medication and initial dosage to minimize the risk of bleeding during surgery or thrombosis postoperatively [53]. Moreover, certain genetic variations can result in poor responses to specific analgesics. For instance, screening for the OPRM1 can predict a patient's response to morphine, enabling tailored pain management strategies that ensure adequate postoperative pain control while avoiding overmedication and associated side effects [54, 55]. Surgeons can use this information to optimize medicine selection, dosage modifications, and reduce the likelihood of adverse drug reactions during the perioperative period, thus improving patient safety and postoperative recovery.

### 3.4 Targeted Therapies

Tumor genetic screening allows surgeons to pinpoint precise molecular targets responsible for cancer growth [56]. For example, in the treatment of NSCLC, patients with EGFR mutations can benefit from EGFR inhibitors like gefitinib or erlotinib, which generally yield fewer side effects than traditional chemotherapy [57]. This knowledge enables the selection of targeted medicines, immunotherapies, or precision surgical procedures suited to the individual's tumor biology, hence increasing treatment efficacy and survival results in cancer patients.

### 3.5 Gene Editing and Gene Therapy

Novel genomic technologies, like CRISPR‐Cas9, enable accurate manipulation of the human genome, providing prospective treatment options for genetic illnesses and hereditary diseases [58]. For instance, studies have shown that dendritic cells edited by CRISPR‐Cas9 can accelerate wound healing via growth factor signaling pathways, significantly altering fibroblast gene expression in the wound bed [59]. A phase 2 trial, identified that NTLA‐2002, a CRISPR‐based vivo gene‐editing therapy, could significantly depress angioedema attacks [60]. Surgeons may employ gene editing techniques in the future to improve wound healing, repair damaged tissues, and correct genetic defects, opening the door to novel surgical procedures and regenerative medicine strategies.

### 3.6 Biomarker Discovery and Prediction

The identification of novel biomarkers linked to the course, prognosis and response to therapy of diseases is made possible by genetic technologies [61]. In prostate cancer, prostate‐specific antigen (PSA) levels enable surgeons to stratify patients by risk, thus guiding the development of tailored treatment plans. Surgeons can also use PSA to detect early tumor residues and recurrences, enabling rapid adjustments to treatment strategies [62]. By incorporating biomarker data into prognostic evaluations, patient counseling, and clinical decision‐making, surgeons can maximize surgical outcomes and guide treatment decisions.

### 3.7 Inherited Disease Management

Genetic testing makes it easier for patients with inherited conditions, like familial polyposis syndromes or hereditary heart diseases, to receive an early diagnosis, family screening, and prompt intervention strategies, like preventive surgery, lifestyle changes or focused surveillance protocols, to reduce the risk of these condition and improve long‐term outcomes. For instance, in managing hereditary heart diseases like Marfan syndrome, detecting mutations in the FBN1 facilitates early diagnosis and monitoring, enabling preventive cardiac surgery when necessary [50, 51]. Effective prenatal monitoring and screening enable early intervention, significantly reducing developmental abnormalities caused by inherited diseases and their long‐term health impacts, thereby lowering societal healthcare burdens.

### 3.8 Genomic Data Integration

Surgeons are empowered with full patient information through the integration of genomic data into electronic health records and surgical decision support systems. This allows for data‐driven clinical insights, risk stratification, and evidence‐based treatment planning customized to each patient's genetic profile.

Leveraging the power of genomic technologies will allow surgeons to lead a new era of precision surgery. This will allow for personalized therapies, optimized outcomes, and proactive management of the underlying genetic factors impacting disease pathology. The amalgamation of genetics and surgery holds great promise for enhancing patient care and influencing surgical practice in the future as genetic research and technology develop.

## 4 Bioengineering Innovations in Tissue Reconstruction

Bioengineering breakthroughs are altering tissue reconstruction by introducing new methods for restoring injured or destroyed tissues [63]. 3/4D printing, tissue engineering, and biomaterials are revolutionizing implant and tissue creation, allowing for more customized solutions with improved biocompatibility and regenerative potential [64, 65]. From difficult organ transplants to face restoration, bioengineering ushers in a new era of accuracy in surgical procedures. These breakthroughs combine biology, engineering, and materials science principles to create biomimetic scaffolds, cell‐based therapeutics, and tissue‐engineering approaches.

### 4.1 Biomimetic Scaffolds

Bioengineers design scaffolds that offer structural support and biochemical signals to facilitate cell attachment, proliferation, and differentiation for tissue and organ repair and reconstruction following trauma or surgery. These scaffolds mimic the native extracellular matrix and can be fabricated into diverse shapes and architectures to meet specific tissue reconstruction requirements. For instance, Chen et al. [66] assessed the efficiency of a biphasic scaffold loaded with autologous cartilage in patients with focal osteochondral lesions. Results indicated that the biphasic scaffold exhibited better clinical outcomes and cartilage refill than marrow stimulation. This advancement represents a significant breakthrough for future surgeries, particularly in cases of extensive tissue damage, creating a new era in the preservation of organ structure and function.

### 4.2 3/4D Bioprinting

Personalized tissue reconstruction and organ transplantation are made possible by 3/4D bio printing technology, which allows bioengineers to create scaffolds and tissue models that are customized to each patient's anatomy [67, 68]. In an RCT, Kesavan et al. [69] developed 3D bio printing‐based extracellular matrix to manage diabetic foot ulcers. Compared with conventional treatment, patients who received a 3D bio printing‐based extracellular matrix showed superior diabetic wound healing. 3D bio printing is increasingly being applied in clinical settings with promising outcomes. Its patient‐specific design and fabrication approach offers superior suitability compared with conventional mass‐produced materials.

### 4.3 Cell‐Based Therapies

Stem cells, including mesenchymal stem cells, induced pluripotent stem cells, and adult tissue‐specific stem cells, hold immense potential for tissue regeneration [70]. Bioengineers harness the regenerative capacity of stem cells by seeding them onto scaffolds or incorporating them into injectable hydrogels, thereby promoting tissue repair, angiogenesis, and remodeling in vivo. Stem cell spheroids with protein/polyphenol self‐assembling armor could induce immunoprotection and immunoregulation to improve myocardial infarction therapies [71]. Zhu et al. [72] developed a microrobot that exhibited natural red blood cell‐mimetic circulation behavior due to its nearly identical surface properties, yet remained magnetically guidable for targeted accumulation at pathological sites, where subsequent laser‐triggered ablation simultaneously released therapeutic payloads and generated localized heat to enhance vascular drug extravasation.

### 4.4 Decellularization and Recellularization

The extracellular matrix architecture and metabolic signals are preserved in donor tissues while cellular components are removed using decellularization procedures. Through the process of recellularization, bioengineers replenish these decellularized scaffolds with patient‐derived cells to produce tissue‐engineered constructions that, whether transplanted or studied in vitro, closely resemble native tissues in terms of both biology and mechanics. For example, Coppi et al. [71] successfully constructed a model of the lung by removing rat lung cells and implanting human lung cells, which can be utilized for various experiments. This strategy establishes a robust foundation for organ cultivation and injury repair while effectively preventing immune rejection through the use of patient‐derived cells.

### 4.5 Gene Therapy

Utilizing gene therapy techniques, therapeutic genes or growth factors are directly delivered to target tissues in order to influence cellular responses, encourage tissue regeneration, and quicken the healing of wounds. Of these, immunotherapy is an outstanding therapy of gene therapy and effectively controls cancer progression [73]. Among these, gene delivery vectors, such as viral vectors or nanoparticles, can improve the effectiveness of gene transfer and regulate the levels of gene expression inside the intended tissue microenvironment.

### 4.6 Organ‐on‐a‐Chip Technology

Organ‐on‐a‐chip systems allow scientists to simulate in vitro tissue interactions, medication reactions, and disease processes by simulating human organ physiological activities inside microfluidic devices [74]. To investigate tissue reconstruction and therapeutic interventions under physiologically realistic settings, bioengineers include perfusable vascular networks, tissue‐specific cells, and biomimetic microenvironments into organ‐on‐a‐chip devices [75]. Organ‐on‐a‐chip systems provide more physiologically relevant experimental conditions than animal models (such as mice), yielding results that better approximate human responses. These systems represent a promising tool for future drug development, efficacy and safety testing, while substantially reducing reliance on animal models.

### 4.7 Bioactive Materials and Drug Delivery Systems

Bioactive materials offer spatiotemporal control over the delivery of bioactive chemicals to target tissues [76, 77]. In tissue reconstruction applications, bioengineers create drug delivery systems with stimuli‐responsive, localized, and sustained release capabilities to maximize therapeutic efficacy and reduce off‐target consequences. For instance, human red blood cell‐derived vesicles could transport lysosomal‐targeted doxorubicin to overcome chemoresistance cancers [78]. Examples of these materials include hydrogels, nanoparticles, and growth factor‐releasing scaffolds. These advanced materials and systems facilitate targeted drug delivery to specific sites or even individual cells, significantly reducing adverse effects while enhancing therapeutic efficacy.

### 4.8 Immunomodulation Strategies

Tissue regeneration and repair depend heavily on immune responses. In order to control the immunological milieu, reduce inflammation, encourage tissue regeneration, and reduce fibrosis and scarring after surgery or injury, bioengineers create immunomodulatory biomaterials and cell treatments. In cancer, many immunomodulation‐based treatments have been applied in clinical practice, such as immune checkpoint inhibitors [79], CAR immune cell therapy [80], and cancer vaccine [81]. These immunomodulatory therapies can be administered either alone or in combination with other treatments [6]. They help establish an immunosuppressive tumor microenvironment to eliminate residual cancer cells and prevent postoperative recurrence, thereby providing effective adjuvant therapy. Additionally, immunomodulators play a significant role in wound healing and tissue regeneration.

Researchers and medical professionals are advancing tissue reconstruction by incorporating these bioengineering advancements, providing promising solutions for replacing and repairing damaged tissues in a variety of clinical scenarios, such as organ transplantation, wound healing, trauma reconstruction, and regenerative medicine therapies.

## 5 Rehabilitation Techniques Redefining Surgical Recovery

Rehabilitation has emerged as an essential component of the surgical framework, optimizing patient outcomes, promoting functional restoration, and improving quality of life after surgical procedures. These procedures take a multidisciplinary approach, combining physical therapy, occupational therapy, assistive technologies, and psychosocial support to meet the different requirements of patients following surgery [82, 83, 84]. Advanced rehabilitation approaches use VI, wearable gadgets, and robotic‐assisted therapies to speed up recovery, improve functional outcomes, and increase patient happiness [85, 86, 87].

### 5.1 Early Mobilization and Physical Therapy

Early mobilization and physical therapy programs aim to reduce postoperative consequences such as muscular weakness, joint stiffness, and respiratory problems by starting rehabilitation activities and ambulation as soon as clinically possible. The ERAS is a comprehensive medical management model aimed at optimizing patient care before, during, and after surgery. Its goal is to minimize surgical trauma and stress responses, shorten recovery times, reduce complication rates, and enhance the quality of surgical recovery [88, 89, 90]. Early Mobilization is an essential component of postoperative rehabilitation, emphasizing the importance of patients initiating activity soon after surgery [88]. Early mobilization helps prevent complications such as pneumonia, deep vein thrombosis, and muscle atrophy. It also aids in restoring muscle function in patients, enhancing circulatory and respiratory system functionality, and promoting recovery [91, 92, 93]. This may involve activities such as standing, walking, and performing simple limb exercises [94]. Physical therapists provide customized exercise programs based on the patient's surgical procedure, functional status, and rehabilitation goals in order to enhance strength, flexibility, and mobility during the healing period. The combination of early mobilization and physical therapy establishes a solid foundation for postoperative recovery, serving as a crucial cornerstone for ensuring surgical efficacy.

### 5.2 Prehabilitation Programs

Prehabilitation programs include focused exercise interventions, dietary counseling, and psychosocial support that are offered prior to surgery to ensure patients' physical and psychological preparedness for the procedure [95, 96]. Prehabilitation programs minimize the incidence of postoperative problems by enhancing preoperative fitness, muscle strength, and overall health. They also speed up recovery and improve surgical outcomes [97, 98, 99]. Despite the long‐standing practice of preoperative smoking cessation, awareness of prehabilitation programs remains limited in clinical preoperative preparation. This necessitates greater surgical team engagement and collaboration with rehabilitation specialists.

### 5.3 Occupational Therapy

Occupational therapists help patients regain independence in activities of daily life after surgery, such as bathing, dressing, cooking, and driving [100, 101, 102]. For example, mental health occupational therapy is a specialized branch of occupational therapy that addresses the unique needs of individuals experiencing mental health difficulties [103]. Occupational therapists in this field work closely with patients to assess their cognitive, emotional, social, and functional abilities, taking into account their personal goals and environmental factors [104]. Through collaborative goal‐setting, therapists help patients engage in activities that are personally meaningful and contribute to their overall well‐being [104, 105]. Occupational therapy therapies aim to improve fine motor skills, cognitive function, and adaptive methods to help patients adjust to life at home and in the community when they leave the hospital.

### 5.4 Supportive Technologies and Flexible Devices

Supportive technologies, such as prosthetics, orthotics, mobility aids, and adaptive equipment, are crucial in helping patients recover functionally and participate in daily activities [106, 107, 108]. Rehabilitation professionals evaluate patients' assistive technology requirements and offer personalized recommendations, training, and changes to maximize device utilization and independence during the rehabilitation process.

### 5.5 Telemedicine and Virtual Rehabilitation

Telemedicine services and virtual rehabilitation platforms allow for the remote provision of rehabilitation therapies, patient progress tracking, and continuous rehabilitation specialist assistance [109, 110]. Tele‐rehabilitation sessions enhance accessibility and continuity of care during the postoperative recovery period by enabling patients to participate in virtual therapy sessions, access personalized exercise programs, and receive real‐time feedback from the comfort of their homes [111, 112]. For example, Li et al. [113]. conducted a feasibility RCT to examine the effects of a home‐based occupational therapy telerehabilitation program via smartphones on functional and motor performance, as well as fall efficacy, in older adults undergoing day hospital rehabilitation after hip fracture surgery in Hong Kong. Results revealed significant improvements in fall efficacy and instrumental activities of daily living performance in the experimental group compared with the comparison group, indicating the potential efficacy of telerehabilitation as an alternative home program for occupational therapy practice with this population. Telemedicine services and virtual rehabilitation platforms enable real‐time guidance, supervision, and correction of rehabilitation training for patients. Their convenience facilitates wider adoption among patients with mild‐to‐moderate functional impairments, eliminating the need for hospital‐based rehabilitation in selected patients while significantly reducing patients' financial burden.

### 5.6 Pain Management Strategies

Pain management techniques include pharmacological interventions, regional anesthesia techniques, and non‐pharmacological modalities like acupuncture [114], mindfulness‐based therapies [115], and transcutaneous electrical nerve stimulation [116]. Comprehensive pain management techniques reduce postoperative pain, promote early mobilization, and improve patients' general comfort and well‐being throughout the healing process.

### 5.7 Psychosocial Support and Counseling

Patients' emotional, social, and psychological needs are met by psychosocial support services, which include peer support groups, stress management, and counselling, all during the surgical recovery process. Psychosocial therapies support emotional well‐being and holistic healing by encouraging resilience, coping mechanisms, and adaptive adjustment to life changes after surgery [117, 118, 119]. The concept of psychosomatic medicine has been widely established. By promoting psychological well‐being, it facilitates physical recovery and supports patients' social reintegration and return to normal daily functioning.

### 5.8 Patient Education and Empowerment

Patient education initiatives enable patients to take an active role in their own healing, comprehend the nature of their surgery, and follow recommended rehabilitation procedures [120, 121, 122]. Patient education programs facilitate autonomy, self‐efficacy, and involvement in rehabilitation activities by offering comprehensive knowledge about postoperative care, self‐management tools, and goal setting. This, in turn, improves surgical recovery results.

## 6 Looking Forward

This work highlights the transformative impact of emerging technologies on surgical advancement. These innovations enable comprehensive perioperative management spanning preoperative assessment, surgical preparation, intraoperative assistance, and postoperative care. AI serves as the central integrator, synergizing genetic technology, bioengineering, and rehabilitation to revolutionize conventional surgical practice. Modern surgery has evolved from a focus on preoperative preparation and operative techniques to a systematic continuum extending from outpatient evaluation to complete postoperative recovery. This paradigm shift presents new challenges and demands for contemporary surgical practitioners. Although the combination of AI, genetic technology, bioengineering, and rehabilitation has great potential, there are significant ethical, legal, and societal issues that need to be addressed. Priorities include eliminating inequities in healthcare delivery, protecting patient autonomy and privacy, and ensuring equal access to technological breakthroughs. Moreover, negotiating the moral, legal, and societal challenges of surgical innovation requires encouraging interdisciplinary cooperation and holding open discussions with stakeholders.

### 6.1 Training Surgeons for the Future

An entirely new approach to surgical education and training is required due to the changing nature of surgery [123]. Three key components of contemporary surgical education—simulation‐based learning, AR platforms, and interdisciplinary collaboration—empower surgeons with the necessary abilities to handle the complexity of next‐generation surgical technologies. Surgical training programs prepare the next generation of surgeons to meet the opportunities and challenges of a constantly changing healthcare landscape by promoting a culture of innovation and lifelong learning. In an education study, AI could pass a medical exam [124]. Furthermore, surgical students improved ergonomics after being trained by an AI‐assisted ergonomics analysis app which could provide immediate feedback to surgical trainees [125]. For instance, an AI‐assisted surgical coaching program could significantly enhance operative performance and safety for novice surgeons performing laparoscopic cholecystectomy by providing comprehensive perioperative support, including preoperative preparation, intraoperative video analysis, and postoperative evaluation [126]. For experienced surgeons, AI can analyze their surgical procedures to identify areas for technique refinement and improvement, providing valuable feedback for skill enhancement [127]. However, additionally, AI could conclude medical research [37]. This function could help surgeons learn knowledge from other fields and quickly understand the synergy of interdisciplinarity.

### 6.2 Reforming Medical Education

Medical education needs to be completely revamped in order to guarantee that technological improvements are seamlessly incorporated into surgical practice. Interdisciplinary training modules covering ethics, legislation, and social ramifications should be incorporated into curricula to develop surgeons who are well‐rounded and able to navigate the ethical, legal, and societal difficulties that come with evolving technologies. Medical education builds a future in which surgical procedures are not only technically sound but also morally and socially just by encouraging a culture of creativity, inclusion, and ethical responsibility [128]. An important aspect is to learn to doubt the correctness of answers. Due to the probability of errors in the answers provided by AI, such errors may harm patients [129]. Therefore, medical education must emphasize cultivating students' spirit of questioning things.

Furthermore, patient education regarding treatment cycle modifications should be implemented through a multi‐phase approach: (1) out‐of‐hospital education via professionally reviewed educational materials; (2) outpatient consultations (in‐person or telemedicine) to explain new treatment protocols; and (3) hospitalization‐phase education using AI‐generated personalized treatment plans. These plans, visualized through modeling technologies (e.g., 3D reconstruction, VR simulations) of the patient's own diagnostic data, enhance comprehension of future treatment pathways while improving physician‐patient communication efficiency.

### 6.3 Challenges and Ethical Considerations

The adoption of genetic technologies, bioengineering and AI in surgery presents several challenges and ethical concerns [130]. AI systems are only as good as the data they are trained on when it comes to the issues of the AI. Surgeons and patients may find it challenging to comprehend the decision‐making processes of AI systems due to their frequent opacity. Relying too much on AI can diminish the surgeon's professional autonomy and judgment. The integration of AI systems like DeepSeek into hospital settings raises concerns about potential limitations on physicians' independent thinking. Moreover, AI may generate incorrect answers, potentially leading to erroneous treatments [129]. Therefore, we argue that regardless of AI's advancement, physicians must maintain independent decision‐making capabilities. To mitigate AI's impact on clinical judgment, several measures can be implemented: (1) requiring AI systems to display detailed reasoning processes, enabling physicians to evaluate the logic and reliability of conclusions while maintaining critical thinking skills; (2) mandating comprehensive citation of sources for all AI‐generated answers to ensure traceability; (3) clearly labeling AI outputs as reference materials rather than definitive answers; and (4) explicitly reminding surgeons that they remain partly responsible for treatment decisions, thereby reinforcing their critical engagement with all diagnostic and therapeutic options. In terms of ethic or legal issues, it may not always be apparent who is ultimately accountable for judgements made using AI, particularly when those decisions have unfavorable consequences. Who is to blame—hospitals, developers, or surgeons [131]? Furthermore, a lot of data is needed for AI systems, and a lot of that data may contain sensitive patient data, which presents serious privacy issues.

The complexity of the mechanics and ramifications of genetic interventions presents a challenge for genetic technology, one that can be challenging for patients and doctors to completely comprehend. Because genetic connections within the human body are complex, genetic alterations may have unanticipated consequences [132]. Consequently, ethical issues include the following [30, 133]: there is a chance that genetic information will be used to discriminate against people in the insurance market, the workplace, or other spheres of life; the possibility of selecting or modifying genetic traits for purposes other than therapy raises ethical questions about eugenics and the commercialization of human genetic modification; and obtaining informed consent is made more difficult by the complex information that patients must understand, including potential risks and benefits. Regarding bioengineering, the difficulties lie in developing tools and materials that blend in with human tissues in a safe and efficient manner while avoiding negative reactions, as well as making sure that organs and tissues that have been bioengineered will continue to be safe and functioning in the long run [134, 135]. The high cost of bioengineering technologies may cause access disparities that favor wealthy patients or nations in the interim. Therefore, there are ethical concerns regarding the moral ramifications of creating and modifying life forms, including the possibility of “playing God” [136]. Additionally, the production and disposal of bioengineered materials may have unanticipated environmental effects.

**FIGURE 1:**
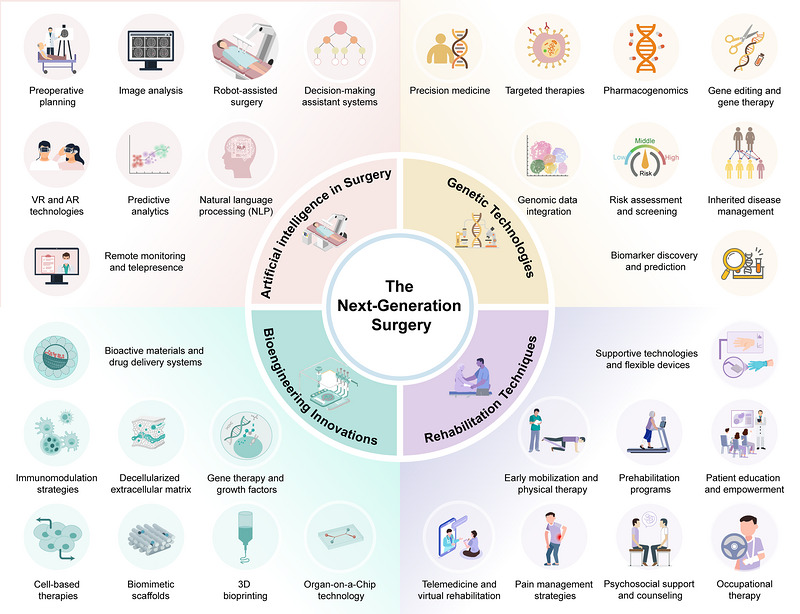
The summary of this article.

All technologies have general ethical considerations, such as making advanced technologies available to all socioeconomic groups to prevent the spread of health disparities; making sure patients are informed about the risks and benefits of new technologies and have the ability to make decisions about their use; creating comprehensive regulations that keep up with technological advancements to ensure patient safety and ethical compliance; and taking into account the possibility of these technologies being misused in non‐medical contexts, which could have serious ethical ramifications [137, 138]. Overall, a coordinated effort involving healthcare practitioners, researchers, policymakers, ethicists, patient advocates, and industry stakeholders is necessary to address these issues and ethical concerns. We can harness the potential of genetic technology and AI to revolutionize surgical care while protecting patient rights, autonomy, and dignity by encouraging discourse, promoting transparency, and preserving ethical norms. Figure 1 exhibited the above perspectives.

## 7 Conclusion

In summary, the integration of cutting‐edge technologies such as AI, genetic technologies, bioengineering, and rehabilitation portends a revolution in surgical operations. Through leveraging innovation and tackling related obstacles, the surgical community may lead the path toward a future where surgical treatment is not just cutting edge in terms of technology but also individualized, compassionate, and all‐encompassing. By working together and exercising ethical stewardship, we can fully utilize next‐generation surgical technologies and bring in a new era of excellence, compassion, and precision in surgical practice.

## Author Contributions

DCF, DXL, JW and ZPW proposed the project, conducted data analysis, interpreted the data, and wrote the manuscript; all authors conducted data analysis, interpreted the data; DCF, KHY, SH, ZHL and WCC supervised the project, and interpreted the data. All authors reviewed and edited the manuscript.

## Funding

The authors have nothing to report.

## Ethical Statement

The authors are accountable for all aspects of the work in ensuring that questions related to the accuracy or integrity of any part of the work are appropriately investigated and resolved.

## Conflicts of Interest

The authors declare no conflicts of interest.

## Data Availability

Data sharing not applicable to this article as no datasets were generated or analysed during the current study.
